# A Machine Learning Approach for Gearbox System Fault Diagnosis

**DOI:** 10.3390/e23091130

**Published:** 2021-08-30

**Authors:** Jan Vrba, Matous Cejnek, Jakub Steinbach, Zuzana Krbcova

**Affiliations:** 1 Department of Computing and Control Engineering, Faculty of Chemical Engineering, University of Chemistry and Technology, Technicka 5, 166 28 Prague, Czech Republic; Jakub.Steinbach@vscht.cz (J.S.); Zuzana.Krbcova@vscht.cz (Z.K.); 2 Department of Instrumentation and Control Engineering, Faculty of Mechanical Engineering, Center of Advanced Aerospace Technology, Czech Technical University in Prague, Technicka Street 4, 166 07 Prague, Czech Republic

**Keywords:** fault diagnosis, gearbox, adaptive filter, NLMS algorithm, support vector machine

## Abstract

This study proposes a fully automated gearbox fault diagnosis approach that does not require knowledge about the specific gearbox construction and its load. The proposed approach is based on evaluating an adaptive filter’s prediction error. The obtained prediction error’s standard deviation is further processed with a support-vector machine to classify the gearbox’s condition. The proposed method was cross-validated on a public dataset, segmented into 1760 test samples, against two other reference methods. The accuracy achieved by the proposed method was better than the accuracies of the reference methods. The accuracy of the proposed method was on average 9% higher compared to both reference methods for different support vector settings.

## 1. Introduction

The gearbox fault detection problem has become an intensively studied topic in the last few decades. Early detection of possible gearbox faults (or rotating machinery in general) can increase the operational safety of a device, as it can reduce the costs of maintenance and prevent total failure. To detect a possible fault of a gearbox, the vibrations of the gearbox are typically measured, often by using multiple sensors at once. The damaged teeth in the gearbox produce force impulses that are usually reflected in the vibration signal. However, evaluating of the vibration signal is difficult. We can view the underlying process of generating the vibration signal as strongly non-linear, non-stationary, and non-Gaussian. The faults produce a signal with energy distributed over various frequencies, which makes successful detection even more difficult. Another issue that arises with the analysis of gearbox vibration signals is that every single gearbox produces a unique signal, so the approach and settings that are successful with some gearboxes may completely fail for different ones. That is probably the reason many other published methods were not validated and evaluated with appropriate datasets. Due to the popularity of this topic, many methods utilizing different approaches have been developed.

As was already mentioned, the signal of a faulty gearbox or rotating machinery has a different spectrum than the faultless one. The fast Fourier transform [1] (FFT) is often used, because it is the most natural tool for studying the frequency spectrum. In the study [2], the authors utilized the DSP-based FFT analyzer that takes advantage of pattern matching techniques to detect faults in a rotating machine. The study [3] evaluated the usability of FFT for predictive maintenance of electrical rotating machines in connection with ISO 2372. In [4] the authors used sparse filtering to extract frequency domain features, classified by the softmax regression classifier, and obtained the output diagnosis results. In [5], the authors suggest using the multiscale chirplet path pursuit algorithm to approximate the best order of the fractional Fourier transform (FrFT) by estimating the instantaneous frequency of the signal component with the largest energy. Then the FrFT spectrum of this component is analyzed and the fault is detected by the frequency sideband evaluation. In [6], the authors combined corrected multiresolution FT with discrete wavelet transform to investigate the vibrations of a gearbox and current transients of a connected DC generator. A comparison of FT and continuous wavelet transform for gearbox fault diagnosis is presented in [7].

There are approaches that are focused on residual signal analysis, such as the utilization of the auto-regressive model [8] or an auto-regressive model with exogenous input [9], where the residual signal is processed and the fault is detected by its features. The study [10] used a neural network to obtain the residual signal which, after Hilbert transform, provided significant information about the gearbox faults.

In some studies, only empirical mode decomposition (EMD) [11] without a Hilbert–Huang transform was used [12,13]. The combination of the Hilbert transform and EMD was presented in the study [14]. The EMD seems an interesting approach to signal evaluation due to its time complexity, as both EMD and FFT have time complexity O(nlogn) [15].

Various methods based on adaptive filtering have been developed over decades. In [16], the authors compared least mean squares (LMS) with linear prediction, spectral kurtosis, and fast block LMS to detect the bearing defect in a gearbox via spectral analysis (note that one of the first uses of LMS in condition monitoring was presented in [17]). They extended their work by comparisons with self-adaptive noise cancellation in [18], and claimed that LMS can, as mentioned in the previous study [16], detect a fault earliest. Another adaptive approach, namely, the adaptive line enhancer, was used in [19]. There were also multiple publications dedicated to the adaptive Schur filter (ASF). The ASF consists of several sections which are described by time-dependent reflection coefficients. Based on the forward prediction error and backward prediction error, the reflection coefficient is calculated for each section [20]. In publication [21], the authors proposed a framework for fault detection based on the changes of the prediction error of the Schur filter. The approach based on monitoring of changes of Schur filter coefficients was presented in [22]. This approach was extended in [23]. An approach to detecting fatigue tooth cracks in a wind turbine gearbox based on the adaptive Morlet wavelet filter was presented in [24]. The study [25] presented a combination of an adaptive noise reducer-based Guassian reference signal technique with the a one-against-one multi-class support vector machine to detect various fault types in a gearbox. The self-adaptive noise cancellation method with nonlinear adaptive filter using a kernel least mean squares algorithm was presented in [26]. Another approach that is based on the adaptive regression splines method and trend change detection was presented in [27]. In [28], the authors proposed a new impulse energy indicator. They utilized an adaptive filter for signal separation, wavelet packet decomposition, and the combination of RMS and kurtosis to select the optimum filter band which indicates the fault in the bearing of the gearbox.

A unique approach based on the estimation of the cointegration factor of a vibration signal to detect the fault of a gearbox was presented in article [29]. Quantitative vibration analysis of bearing faults is exhaustively presented in [30], where the authors present a dynamic model of rolling element bearings and provide simulation results for a specific fault.

Recently, multiple methods using deep learning have emerged. In [31] the authors proposed to use an augmented deep sparse autoencoder to process the raw vibration signal. Study [32] avoided the need for a large dataset to teach a deep learning model by using a stacked sparse autoencoder that processes time-frequency images. Another approach that was presented in [33] uses multimodal deep support vector classification in combination with a Gaussian–Bernoulli deep Boltzmann machine. In [34], the authors combined improved particle swarm optimization, variational mode decomposition, and an improved convolutional neural network to process a signal spectrum and composite fault signal. In [35] the authors processed vibration, acoustic, and torque signals via discrete wavelet transform to obtain initial features for deep neural networks. The usage of convolutional neural networks was also presented in [36]. Acoustic-based diagnosis based on a multiscale convolutional learning structure and an attention mechanism was presented in [37]. An interesting approach based on image processing was introduced in [38], where images with signal frequency spectra obtained via variational mode decomposition are used as inputs for a convolutional neural network. The study [39] presented the use of a deep random forest fusion technique to fuse acoustic emission and vibratory signals to detect various gearbox faults. In [40], the authors compared long-short-term memory and bi-directional long-short-term memory (LSTM) models for gearbox health monitoring. In [41], the authors transformed the vibration signal into an image-like simplified health data map that visualized a tooth-wise fault of the gearbox. This image was then processed by a convolutional neural network, and the remaining domain shift problem was solved via maximum classifier discrepancy. A diagnostic method based on time-frequency representation and deep reinforcement learning was presented in [42]. A diagnostic method based on bidirectional convolutional LSTM networks is presented in [43]. The authors claimed that their architecture can solve the problem of extracting spatial and temporal features simultaneously without losing any information. The study [44] introduced 1D deep convolutional transfer learning to process a torque measurement and estimate the health state of the gearbox. A deep morphological convolutional neural network for vibration signal processing was introduced in the article [45]. A new special type of CNN—the multiscale fusion global sparse network—for gearbox fault diagnosis, was proposed in [46]. Another unique neural network architecture, AKRnet, utilizing attentive kernel residual learning for feature learning of gearbox vibration signals, was presented in [47]. In [48], the authors proposed a fault diagnosis system that combines ResNet [49] with wavelet tranform, and showed that their hybrid attention-based method improves ResNet’s performance. A novel deep neural network which combines EMD, LSTM, and particle swarm optimization was presented in the study [50]. A method based on the usage of a two-class nonnegative matrix factorization network was proposed in [51]. There are many more applications of deep learning techniques in gearbox fault diagnosis that were published recently, which we are aware of but not mentioning here due to the scope of this article. In the study [52], the authors applied self-organizing maps with kurtosis criterion obtained via variational mode decomposition.

More information about gearbox fault detection approaches can be found in the following review papers: [53,54,55].

Most of the studies mentioned above were mainly qualitative, and some of their methods require expert opinions to conclude on the gearbox’s condition. However, in our study, we focused on a simple, robust, and fully automated solution of gearbox fault detection without prior knowledge of the operation or measurement details, and without a need for a large training dataset. Those factors make it different to many of the other deep learning-based methods. The proposed method features a multiscale approach and can utilize a custom number of parallel sensors attached to the gearbox. The evaluation criteria were assessed using measurement data, and the proposed approach was cross-validated.

## 2. Materials and Methods

Our proposed method is based on utilizing the error prediction of an adaptive filter. The description of the adaptive filter is presented in Section 2.2. The adaptive filter coefficients are adapted via NLMS algorithm (see Section 2.3). Detailed description of the proposed descriptor is in Section 2.4. This descriptor is evaluated via support vector machine (SVM) and 10-fold cross-validation is performed (see Section 2.6 and Section 2.7). Information about the used dataset is provided in Section 2.1 and overall experiment description in Section 2.8. Results of the proposed approach are compared with multiple methods in Section 3, those reference methods are introduced in Section 2.5.

### 2.1. Dataset

A publicly available dataset already presented in the paper [56] was used in this work. The dataset includes vibration measurements from healthy and broken gearboxes under various loads and a constant rotating speed at 30 Hz. The measurements were recorded via SpectraQuest’s Gearbox Fault Diagnostics Simulator.

The original whole data $x_{s}$ contain a time series of various lengths. This unbalance was corrected with data segmentation. The original dataset was segmented into K=1000 data point-long segments. For every condition (broken/healthy) and every load (0–90% with step 10%), P=88 segments were formed. Leftover data from the longer time series were not used. The resulting balanced dataset consists of 1760 segments. Every segment contains J=4 time series measured by sensors placed on different places (4 sensors). Therefore, the data segment can be described as

(1) $$x_{j}^{p}=\left[x_{sj}\left(p\cdot K-K\right),x_{sj}\left(p\cdot K-K+1\right),\cdots ,x_{sj}\left(p\cdot K-1\right)\right]$$

Note that index j∈{1,2,3,4} represents the sensor ID and p∈{1,2,⋯,N} is the segment number. Note the total number of segments available N=1760 is:

(2) $$N=P\cdot n_{condition}\cdot n_{loading}$$

where $n_{condition}=2$ (broken, healthy) and $n_{loading}=10$ (loading from 0 to 90% with 10% step).

According to our results, every sensor provides a different amount of information about the health status of the gearbox. Furthermore, the information from the independent sensors is not fully correlated, and thus the sensors can be used in a complementary fashion.

### 2.2. Adaptive Filter

**Figure 1:**
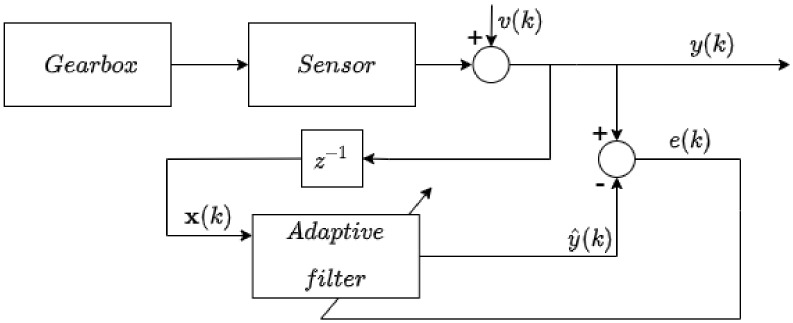
Block schema of adaptive filtration process.

The scheme that depicts the adaptive filtration problem is in Figure 1, where x(k)∈R is the input signal, v(k)∈R represents additive noise, y(k)∈R is measured output of the system, $\hat{y}\left(k\right)\in R$ is the output of the adaptive filter and e(k)∈R represents the error of the filter (prediction error). In our proposed method, we have used the linear adaptive filter with finite impulse response. The output of the filter at discrete time index k∈Z is given by

(3) $$\hat{y}\left(k\right)=\sum_{i=0}^{N}w_{i}\left(k\right)x\left(k-i\right)$$

where $w_{i}\left(k\right)\in R$ is the value of the *i*-th adaptive weight, N+1 is the number of adaptive weights and x(k−i) represents the delayed sample. The output of the FIR filter (Equation (3)) in vector notation can be written as

(4) $$\hat{y}\left(k\right)=x{\left(k\right)}^{T}\cdot w\left(k\right)$$

where w(k) is the vector of adaptive weights given as

(5) $$w\left(k\right)=[w_{0}\left(k\right),\cdots ,w_{N}\left(k\right)]$$

and x(k) represents the input vector given as

(6) x(k)=[x(k),⋯,x(k−N)].

Note that the adaptive weights were updated via NLMS algorithm (Section 2.3) with every sample obtained.

**Figure 2:**
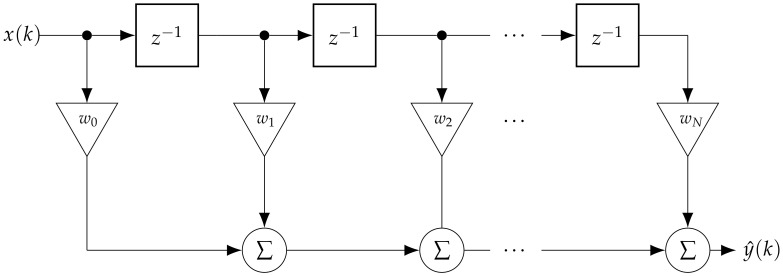
Block schema representation of a digital adaptive filter.

The block schema of the filter is depicted in Figure 2. The block with $z^{-1}$ represents unit time delay (if we consider *Z*-transform notation).

### 2.3. The NLMS Algorithm

The proposed method is based on the error evaluation of a normalized-last-mean-square (NLMS) adaptive filter [57] in predictive settings.

The NLMS algorithm is a modification of the LMS (stochastic gradient descent). The LMS weight adaptation is given as follows.

(7) w(k+1)=w(k)+Δw(k),

where Δw(k) is

(8) $$\Delta w\left(k\right)=\frac{1}{2}\mu \frac{\partial e^{2}\left(k\right)}{\partial w(k)}\;=\mu \cdot e\left(k\right)\cdot x\left(k\right),$$

where μ∈R is the learning rate (step size) and e∈R is error, which is defined as

(9) $$e\left(k\right)=y\left(k\right)-\hat{y}\left(k\right).$$

The NLMS adaptation rule is given as follows:

(10) Δw(k)=η(k)·w(k)·e(k),

where η(k) is an actual learning rate normalized with ${\left||x\left(k\right)|\right|}^{2}$ (input signal power) as follows:

(11) $$\eta \left(k\right)=\frac{\mu }{\epsilon +{\left||x\left(k\right)|\right|}^{2}}.$$

where ϵ∈R is a small positive constant (regularisation term) introduced to preserve stability for inputs close to zero. The NLMS with ϵ is also called ϵ-NLMS. The NLMS algorithm is stable if

(12) $$0\leq \mu \leq 2+\frac{2\epsilon }{{\left||x\left(k\right)|\right|}^{2}},$$

or for the case without a regularization term: ϵ

(13) μ∈〈0,2〉.

The optimal learning rate is affected by the properties of additive noise v(k). In the case that the additive noise is uncorrelated with input signal x, the optimal learning rate is given as

(14) $$\mu_{optimal}=\frac{E[{\left|\hat{y}\left(k\right)-\tilde{y}\left(k\right)\right|}^{2}]}{E[{\left|e(k)\right|}^{2}]}$$

The adaptive filter prediction error is obtained for every predicted data point in step-by-step predictive settings. This operation is done for every sensor independently. In this case, an input of the filter x(k) consists of previously measured values y as follows:

(15) x(k)=[y(k−1),…,y(k−n)].

where the filter length *n* directly represents the number of historical values used for prediction. In this study, the filter size was set to n=10, because this setting yielded the best results.

### 2.4. Proposed Descriptor

To detect a gearbox fault, we propose a descriptor based on the prediction error of the adaptive filter adapted via NLMS algorithm. This data segment $x_{j}^{p}$ is processed by an adaptive filter that utilizes NLMS algorithm to obtain the filter prediction error $e_{j}^{p}$. Assume the corresponding segment of filter prediction error as

(16) $$e_{j}^{p}=\left[e_{j}^{p}\left(0\right),e_{j}^{p}\left(1\right),\cdots ,e_{j}^{p}\left(K\right)\right]$$

The resulting descriptor for the whole segment of filter error $e_{p}^{j}$ (9) is evaluated as follows.

(17) $$c_{error}=\sqrt{E\left[{\left(e_{j}^{p}\right)}^{2}\right]-{\left(E[e_{j}^{p}]\right)}^{2}},$$

where *E* stands for the expected value. This descriptor represents the actual effort of the adaptive filter made to follow the target signal. This effort can be understood as a novelty in the data, or irregularity in the measured signal. Relation between irregularity in the data and the health condition of the gearbox is the main idea behind the proposed method. Note that in our study we processed only nonoverlapping segments.

### 2.5. Reference Methods

To present a suitable challenge for the proposed method, two other reference methods were used to evaluate the health status of the gearbox.

#### 2.5.1. Standard Deviation of the Raw Data

The first reference method is based on evaluation of the raw measured data standard deviation as the direct feature for the gearbox health assessment:

(18) $$c_{plain}=\sqrt{E\left[y^{2}\right]-{\left(E\left[y\right]\right)}^{2}}.$$

This method represents the most intuitive and computationally cheap way to measure irregularities in data. However, this approach is usable only if the standard deviation represents the underlying distribution.

#### 2.5.2. Standard Deviation of the First IMF

**Figure 3:**
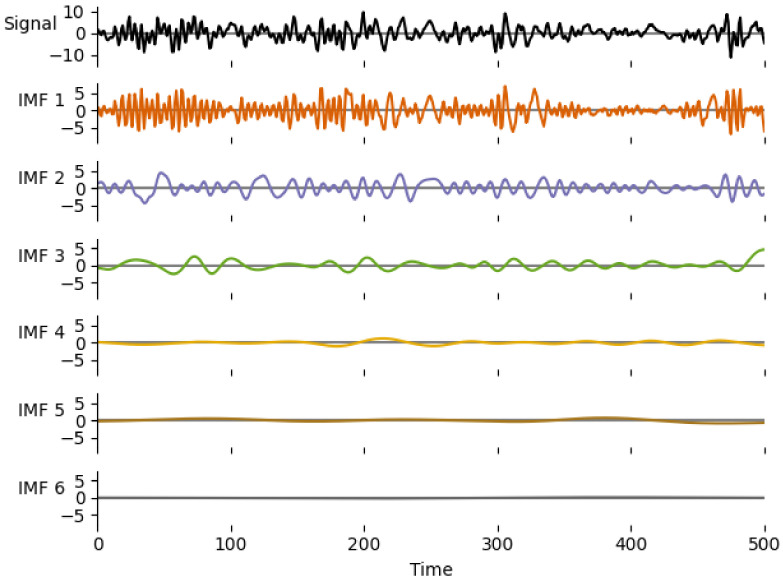
Resulting IMF of a gearbox signal sample. Note: an IMF is not a harmonic wave and the higher IMFs contain waves with generally lower frequency—the last IMF represents the slowest trend in the signal.

**Figure 4:**
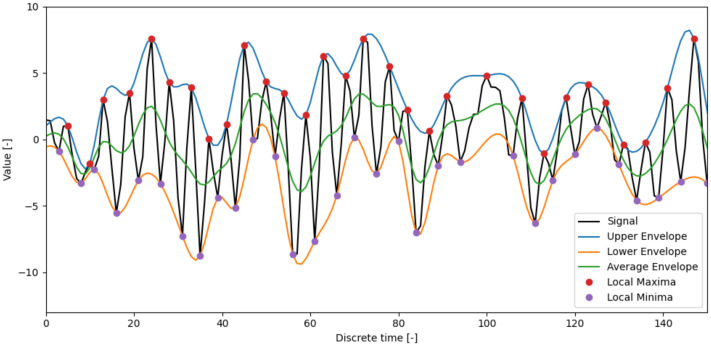
One step of the sifting process applied to a gearbox signal sample. Upper and lower envelopes are cubic splines connecting the local extremes.

Various frequency analysis methods are common tools for diagnosing faults in rotating machinery. Therefore, the second reference method used is based on frequency analysis of data. Due to the nonstationary nature of the gearbox vibration measurements, the Hilbert–Huang transform (HHT) [58] is used more often than the Fourier transform (FT) [1]. The fundamental part of the HHT is the empirical mode decomposition (EMD) method [11]. The EMD breaks down signals into components. In some studies, the EMD without the HHT is used [12,13]. Using the EMD method, any complicated dataset can be decomposed into a finite and often small number of components—intrinsic mode functions (IMFs). Any IMF represents a generally simple oscillatory mode that can be understood as a counterpart to the simple harmonic function. An IMF is any function with the same number of extrema and zero crossings, whose envelopes are symmetric with respect to zero. An example of a gearbox signal’s sample IMFs is presented in Figure 3. The process of an IMF extraction is called sifting. The sifting process can be described as:

1. Create upper and lower envelope—connect local minima/maxima by a cubic spline line.
2. Get the first component ($h_{1}$) from original signal and mean value ($m_{1}$) of lower and upper envelopes:
  (19) $$x-m_{1}=h_{1}.$$
  The envelopes and local extremes of a gearbox signal sample are shown in Figure 4.
3. Repeat the previous step *i* times:
  (20) $$h_{1(i-1)}-h_{1i}=h_{1i}.$$
4. Obtain the first IMF
  (21) $${\mathrm{imf}}_{1}=h_{1(i)}$$
5. Repeat previous steps to get other IMFs.

The number of sifting steps (*j*) is determined via the stoppage criterion. There are four known stoppage criteria for the sifting process: the standard deviation criterion; the number criterion; the threshold method; energy difference tracking. The implementation of EMD used in this study utilizes the standard deviation criterion. The sifting process stops when the standard deviation is smaller than a pre-supplied value. The standard deviation of the sifting step is determined as [40]:

(22) $$SD_{i}=\sum_{k=0}^{K}\frac{|h_{i-1}\left(k\right)-h_{i}{\left(k\right)|}^{2}}{h_{i-1}^{2}\left(k\right)}.$$

In this work, the EMD was used to filter out the slower frequencies from the data to enhance the standard deviation change present in the original data. Then, the standard deviation was evaluated utilizing the first intrinsic mode function (IMF):

(23) $$c_{emd}=\sqrt{E\left[{\mathrm{imf}1}^{2}\right]-{\left(E\left[\mathrm{imf}1\right]\right)}^{2}}.$$

The first IMF was chosen because it had the greatest influence on the final accuracy in this given experiment setup. This frequency-based data selection enhances the irregularity related to the gearbox condition and thus improves any evaluation.

### 2.6. Decision Making via SVM

All studied methods (proposed and reference ones) extract a single scalar (standard deviation) as the descriptor of the whole sensor measurement. However, the measured data contain four different sensor measurements. Every sensor contains slightly different information. To make a prediction on gearbox health state from those 1–4 available descriptors, the SVM [59] is used. The used SVM dataset of *k* points was formed as

(24) $$\left(d_{1},l_{1}\right),\ldots ,\left(d_{k},l_{k}\right),$$

where every $d_{i}$ is a vector of 1–4 data segment descriptors and $l_{i}$ is the data segment class (healthy/faulty). Assume the hyper-plane that divides the group of points into two classes, takes the form

(25) $$a^{T}d+b=0,$$

Assume that the support vector equations representing the healthy/faulty class are given as

(26) $$-a^{T}d+b\geq \alpha \;\mathrm{for}\;l_{i}==+1$$

(27) $$-a^{T}d+b\leq \alpha \;\mathrm{for}\;l_{i}==-1$$

where +1 and −1 represent the healthy class and faulty class. To find the optimal hyper-plane, we have to solve the following minimization problem:

$$\min_{a,b}\frac{1}{2}{∥a∥}^{2}$$

(28) $$\mathrm{subject}\mathrm{to}l_{i}\left(a\cdot x+b\right)-1\geq 0,\mathrm{for}i=1,\cdots ,k$$

Note that $\frac{b}{∥a∥}$ determines the offset of the hyper-plane from the origin along the normal vector a. A linear kernel is used in the SVM algorithm implementation [60].

### 2.7. N-Fold Cross-Validation

**Figure 5:**
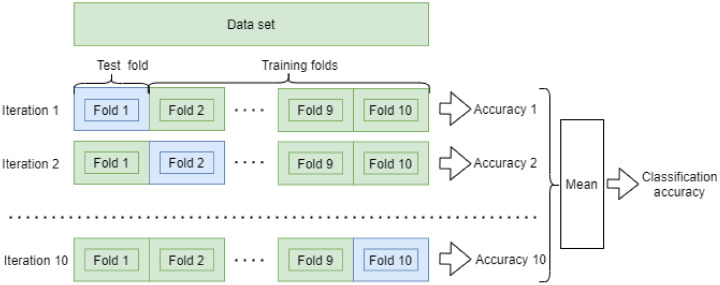
Schematics of the 10-fold cross-validation process used in this study.

Exhaustive 10-fold cross-validation was used to obtain the accuracies of the proposed and reference methods. Before splitting the data into ten same size chunks, the segments were shuffled to balance the healthy vs. broken segment ratio in every chunk. In every iteration of cross-validation, one chunk was used for testing and nine chunks were used for training. The average accuracy was determined over all testing chunks. The scheme of the cross-validation process is depicted in Figure 5.

### 2.8. Experimental Framework Overview

**Figure 6:**
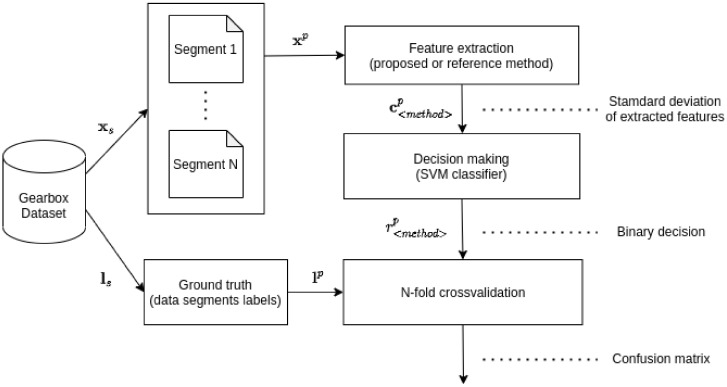
Experimental design—from raw data to final accuracy measure.

The core of the proposed method is the approach of extracting features from the raw data. The extracted features can be evaluated with any classifier. In this study, the well-known support vector machine (SVM) [59] was used. The classification outputs (gearbox condition) of the proposed method and the reference methods were validated via n-fold cross-validation. An overview of the experiment is displayed in Figure 6.

**Figure 7:**
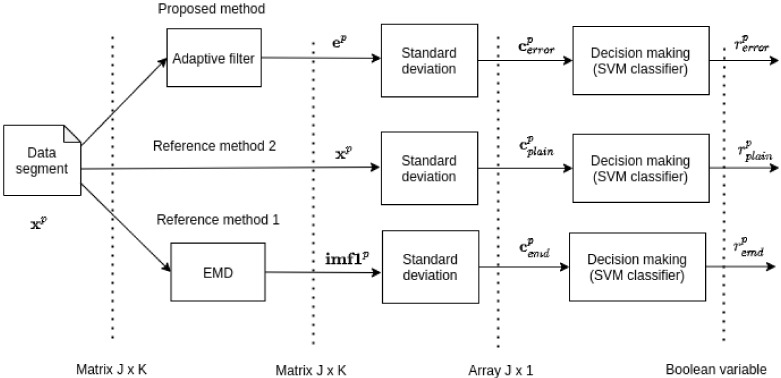
Experimental design—data flow and shapes in the experimental framework.

In this study, we compare the proposed method with two reference methods. The data shapes and organization of the methods in the testing framework are displayed in Figure 7.

## 3. Results

**Figure 8:**
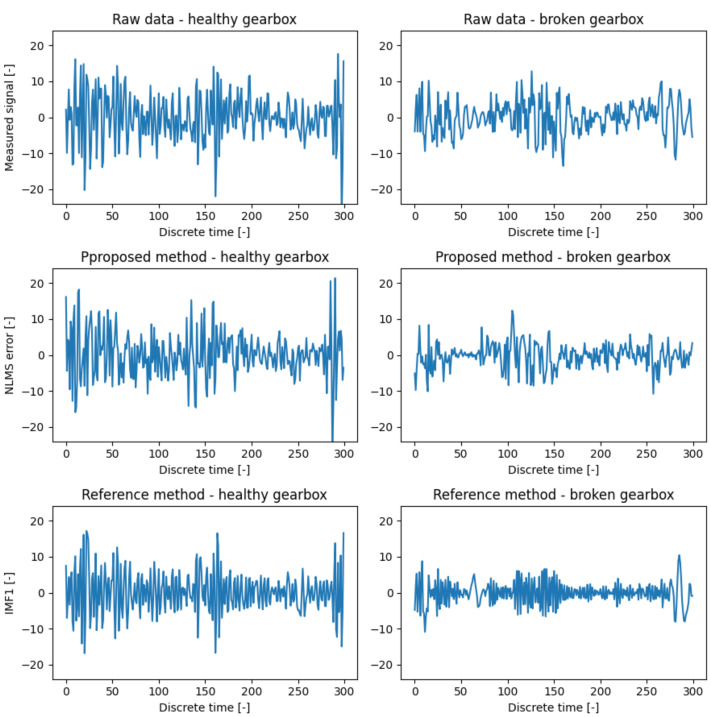
Comparison of features used for proposed and reference methods.

The full cross-validation results are shown in Table 1. Generally, the best results were achieved by the proposed method. The second best method was the one based on EMD filtration, and the worst results were achieved via evaluation of the raw data (denoted as PLAIN in the table). An interesting exception was the combination of channels 3 and 4, where the PLAIN method outperformed the EMD based method. An illustrative example of the extracted features is in Figure 8.

**Figure 9:**
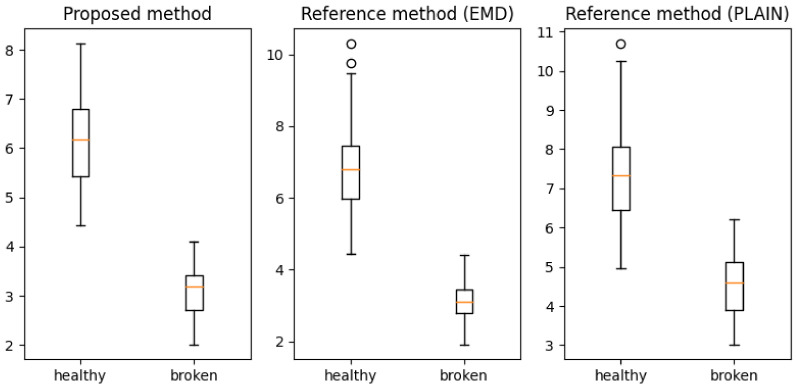
Boxplots of fault descriptors extracted by proposed and reference methods for sensor 1 (the most information).

**Figure 10:**
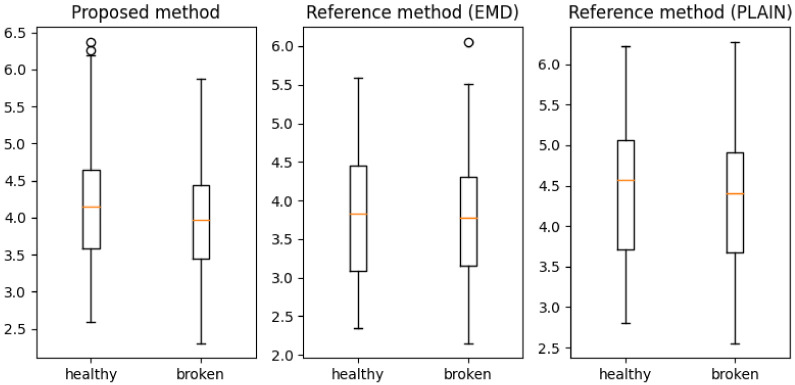
Boxplots of fault descriptors extracted by proposed and reference methods for sensor 4 (the least information).

Furthermore, a method’s consistency, shown in Table 1, can be used to estimate the amount of fault-related information in each sensor’s time series. Sensor 1 provided the greatest amount of useful information and dominated the prediction accuracy, and sensor 4 provided the least. To clarify this observation, box plots of the accuracies achieved from sensor 1 (Figure 9) and sensor 4 are shown (Figure 10).

Moreover, note that a higher number of sensors increases the accuracy.

## 4. Discussion

A novel and time-effective method of gearbox fault detection was proposed in this paper. The proposed method is superior in comparison to the reference methods: raw data statistics and frequency analysis-based feature extraction. Furthermore, the proposed method does not need any information about the measurement setup or the gearbox. The fault detection is based purely on machine learning. The classification criteria are determined according to the data. In other words, the proposed approach can adapt the gearbox diagnosis criteria according to the particular gearbox without human expert input or supervision.

The ultimate goal of this paper was to show that the adaptive filtration error can enhance the information in raw data (the first reference method) in a greater way than the other often-used method of feature extraction—EMD (the second reference method). Therefore, we used a straightforward, noncomplex approach of standard deviation estimation and SVM classification for simple and robust evaluation of the methods. More complex evaluation methods can lead to misinterpreting the feature extraction methods.

The possible utilization of a more advanced classifier (such as neural network) would be a suitable future direction for the research of the proposed feature extraction approach. Another attractive future study might be on applicability research regarding different fault detection tasks, including rotating machinery.

## Figures and Tables

**Table 1:** Resulting accuracies of the methods; 100% means the best accuracy, and 50% is equal to classification by a fair coin-flip.

Sensors (SVM Inputs)	Classification Accuracy [%]		
	Proposed Method (NLMS Error)	Reference Method (EMD—IMF 1)	Reference Method (PLAIN)
1, 2, 3, 4	100.0	100.0	100.0
2, 3, 4	93.58	71.534	72.727
3, 4	71.875	58.58	71.875
4	55.114	50.852	52.33
3	65.398	52.67	58.92
2	81.818	71.25	54.886
1	100.0	99.886	94.886

## Data Availability

The dataset we used is publicly available at the address: https://openei.org/datasets/dataset/gearbox-fault-diagnosis-data (accessed on 20 August 2021).

## Abbreviations

The following abbreviations are used in this manuscript:

ASF	adaptive Schur filter
CNN	convolutional neural network
EMD	empirical mode decomposition
FrFT	Fractional Fourier Transform
FT	Fourier transform
HHT	Hilbert–Huang transform
IMF	intrinsic mode function
LMS	least mean squares
LSTM	long short-term memory
NLMS	Normalized least mean squares
SVM	support-vector machine
